# Characterization of the complete mitochondrial genome of Tibet Gaoshan Yak (*Bos grunniens*)

**DOI:** 10.1080/23802359.2021.2001389

**Published:** 2021-11-23

**Authors:** Wenwen Ren, Qiang Zhang, Renqing Dingkao, Chun Huang, Xingdong Wang, Zhenyu Zhang, Hui Jiang, Xian Guo, Ping Yan, Chunnian Liang

**Affiliations:** a Key Laboratory of Yak Breeding Engineering of Gansu Province, Lanzhou Institute of Husbandry and Pharmaceutical Sciences, Chinese Academy of Agricultural Sciences, Lanzhou, China; b Life Science and Engineering College of Northwest Minzu University, Lanzhou, China; c Institute of Husbandry and Pharmaceutical Sciences, Tebet Academy of Agricultural and Animal Husbandry Sciences, Lhasa, China; d State Key Laboratory of Hulless Barley and Yak Germplasm Resources and Genetic Improvement, Lhasa, China

**Keywords:** Tibet Gaoshan Yak, mitochondrial genome, phylogenetic analysis

## Abstract

Tibet Gaoshan Yak (*Bos grunniens*) is a local Yak breed that mainly produces meat in Tibet Autonomous Region, China. In this study, the complete mitochondrial genome of Tibet Gaoshan Yak was sequenced. The total length of the mitochondrial genome is 16,323 bp, and the base composition is 33.71% for A, 13.21% for G, 27.27% for T, and 25.81% for C. The genome includes 13 protein-coding genes (*ND1-ND6*, *ND4L*, *COX1-COX3*, *ATP6*, *ATP8,* and *CYTB*), two rRNA genes (12S rRNA and 16SrRNA), 22 tRNA genes, and a noncoding control region (D-loop). Phylogenetic analysis showed that Tibet Gaoshan Yak has the closest relationship with Polled Yak. The sequence analysis provided in this study will be helpful to the management of Yak breeds, the origin, and evolution of Yak, and the protection and utilization of genetic resources.

China has the largest number of Yaks in the world (Wiener et al. 2011). Tibet Gaoshan Yak is a local Yak breed in China, mainly produced in the alpine grassland of the eastern Tibet Autonomous Region. Tibet Gaoshan Yak has strong adaptability to the extreme environment of high altitude, low oxygen content, large temperature difference and short growing period of forage in its distribution area. It is an important animal species indispensable for local people's production and life (Huang et al. 2012). However, up to now, there is no report on the complete sequence of mitochondrial genome in Tibet Gaoshan Yak. Mitochondrial DNA is a circular molecule and it is a powerful molecular tool in evolutionary and phylogenetic studies (Wang et al. 2011); this study reported the complete mitochondrial DNA sequence of Gaoshan Yak also in comparison with other breeds.

In this study,blood samples of Tibet Gaoshan Yak were collected from Baqing County, Naqu City, Tibet Autonomous Region (N31. 924638, E94. 059203), preserved at −20 °C in the Key Laboratory of Yak Breeding Engineering of Gansu Province, Lanzhou Institute of Husbandry and Pharmaceutical Sciences (Lanzhou, Gansu Province, China) with a storage number: LZ2020-008. Referring to the manufacturer’s instructions, total genomic DNA was extracted from 20 μl of blood by using Easy Pure Blood Genomic DNA Kit (Transgen Biotch, Beijing, China). Six pairs of primers (Table 1) (Wang et al. 2011) were used to amplify mitochondrial DNA by polymerase chain reaction. The thermocycler settings were as follows: 95 °C for 3 min; 35 cycles of 95 °C for 20 s, 52 °C for 40 s, and 72 °C for 4 min, followed by 72 °C for 10 min. PCR products were detected by 1 % agarose gel electrophoresis. PCR products with appropriate band size were then sent to Xi’an Qinco Biotechnology Co., Ltd (Xi’an, China) for sequencing. The sequencing results were assembled by using DNASTAR 7.1 (Burland 2000) software.

**Table 1.** Primers used for amplifying the complete mitochondrial genome.

Primer set	Sequence (5’→3’)	PCR product size (bp)
1st pair	AAATGACGAAAGTGACCCTA	2688
	TAGGGCTCCGATTAGTGCGT	
2nd pair	CCTACGTGATCTGAGTTCAG	4190
	TGAGCCCATTGATGAGACAG	
3rd pair	TCTACTATTTGGAGCCTGGG	3942
	CGAAATGTCAGTATCAGGC	
4th pair	AAGCCCTTGACCCCTTACAG	2795
	TCGTGTAAAGGAAGGTGAGA	
5th pair	CAAACGGACCTAAAATCACT	3293
	GTGTATTGCTAGGAATAGGC	
6th pair	AGAAAACCCTACGAAACCAA	3523
	CTTTCATCGTTCCCTTGCGG	

The complete mitochondrial genome of Tibet Gaoshan Yak was determined and deposited in GenBank with an accession number: MT916767. The mitochondrial genome of the Tibet Gaoshan Yak was a circular molecule with 16,323 bp in length, and the composition of the overall nucleotide is 33.71% for A,13.21% for G,27.27% for T and 25.81% for C, including 13 protein-coding genes (*ND1-ND6*, *ND4L*, *COX1-COX3*, *ATP6*, *ATP8* and *CYTB*), two rRNA genes (12S rRNA and 16S rRNA), 22 tRNA genes and a noncoding control region (D-loop). The gene sequence and composition of the mitochondrial genome of the Tibet Gaoshan Yak is similar to other Yak species (Huang et al. 2019). The overlap of ATPase genes seemed to be a common phenomenon in most vertebrate mitochondrial genomes (Li et al. 2014). We found three overlaps in the protein-coding genes of this sequence,including ATP6 overlaps with ATP8 for 40 bp, ND4 overlaps with ND4L for 7 bp and ND5 overlaps with ND6 for 17 bp. The alignment of these genes is conservative compared to other Yak subspecies and Bovidae.

Based on 18 complete bovine mitochondrial genomes, we constructed a phylogenetic tree using MEGA6.0 (Tamura et al. 2013) software combined with the Maximum Likelihood (ML) method of 1000 bootstrap replicates. The tree with the highest log likelihood (−43,542.1851) is shown in Figure 1. The percentage of trees in which the associated taxa clustered together is shown next to the branches. Initial tree(s) for the heuristic search were obtained automatically by applying Neighbor-Join and BioNJ algorithms to a matrix of pairwise distances estimated using the Maximum Composite Likelihood (MCL) approach, and then selecting the topology with superior log likelihood value. A discrete Gamma distribution was used to model evolutionary rate differences among sites (5 categories (+*G*, parameter = 0.8747)). The rate variation model allowed for some sites to be evolutionarily invariable ([+*I*], 55.9107% sites). The tree is drawn to scale, with branch lengths measured in the number of substitutions per site. The analysis involved 18 nucleotide sequences. Codon positions included were 1st + 2nd + 3rd + Noncoding. All positions containing gaps and missing data were eliminated. There were a total of 16,275 positions in the final dataset. The molecular phylogenetic analysis revealed that 18 species are divided into two branches as a whole and Tibet Gaoshan Yak has a closer genetic relationship with Polled Yak (Figure 1). In conclusion, the complete mitochondrial genome sequence of Tibet Gaoshan Yak can be used to further study of its origin evolution and breeding improvement.

**Figure 1.**
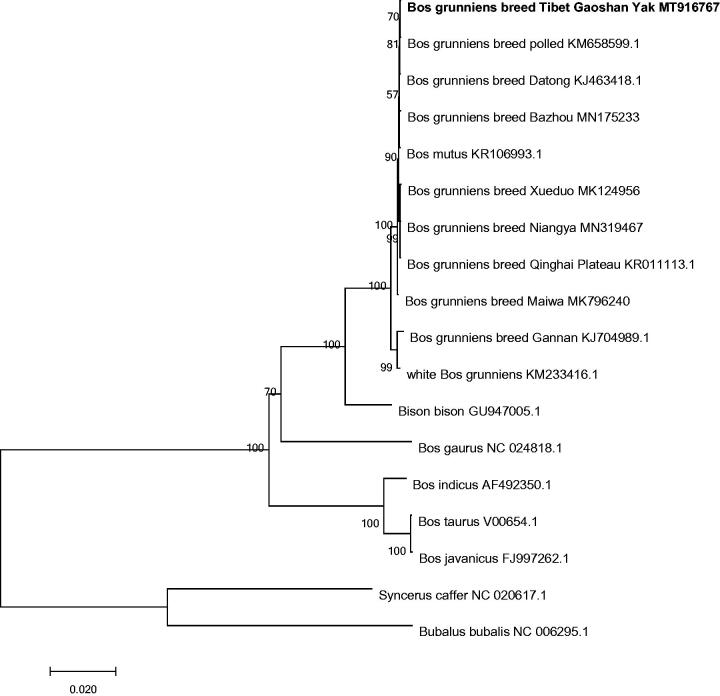
Molecular phylogenetic analysis by maximum likelihood method.

## Data Availability

The data that support the findings of this study are openly available in GenBank of NCBI at https://www.ncbi.nlm.nih.gov, reference number MT916767.
